# Hydroxybenzoate hydroxylase genes underlying protocatechuic acid production in *Valsa mali* are required for full pathogenicity in apple trees

**DOI:** 10.1111/mpp.13119

**Published:** 2021-08-13

**Authors:** Lulu Meng, Cuicui Sun, Liyong Gao, Muhammad Saleem, Baohua Li, Caixia Wang

**Affiliations:** ^1^ College of Plant Health and Medicine Key Laboratory of Integrated Crop Pest Management of Shandong Province Shandong Province Key Laboratory of Applied Mycology Qingdao Agricultural University Qingdao China; ^2^ Department of Biological Sciences Alabama State University Montgomery Alabama USA

**Keywords:** apple trees, hydroxybenzoate hydroxylase, pathogenicity, toxin, *Valsa mali*

## Abstract

*Valsa mali* is the causative agent of apple tree valsa canker, which causes significant losses in apple production. It produces various toxic compounds that kill plant cells, facilitating infection. Among these, protocatechuic acid exhibits the highest phytotoxic activity. However, those genes involved in toxin production have not been studied. In this study we identified four hydroxybenzoate hydroxylase genes (*VmHbh1*, *VmHbh2*, *VmHbh3*, and *VmHbh4*) from the transcriptome of *V*. *mali*. The VmHbh protein had high enzymatic activities of hydroxybenzoate hydroxylase, which could convert 4‐hydroxybenzoate to protocatechuic acid. These four *VmHbh* genes all had highly elevated transcript levels during the *V*. *mali* infection process, especially *VmHbh1* and *VmHbh4*, with 26.0‐ and 53.4‐fold increases, respectively. Mutants of the four genes were generated to study whether *VmHbh*s are required for *V*. *mali* pathogenicity. Of the four genes, the *VmHbh1* and *VmHbh4* deletion mutants considerably attenuated *V*. *mali* virulence in apple leaves and in twigs, coupled with much reduced toxin levels. The *VmHbh2* and *VmHbh3* deletion mutants promoted the transcript levels of the other *VmHbh*s, suggesting functional redundancies of *VmHbh*s in *V*. *mali* virulence. The results provide insights into the functions of VmHbhs in the production of protocatechuic acid by *V*. *mali* during its infection of apple trees.

## Graphical abstract

Hydroxybenzoate hydroxylase genes of *Valsa mali* contribute to full pathogenicity during pathogen infection apple trees by regulating protocatechuic acid production.

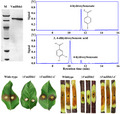

## 1 INTRODUCTION

*Valsa mali* is a necrotrophic fungal pathogen that mainly infects branches and trunks of apple trees, causing significant losses to apple production in eastern Asia, especially in China (Li et al., 2013; Wang et al., 2012). It is characterized by abundant pycnidia on cankers that can release conidia throughout the year (Li et al., 2013). This pathogen infects apple trees with conidia through wounds, and may rapidly expand into the xylem, making its control difficult (Abe et al., 2007; Chen et al., 2016). To date, our strategies to effectively prevent and control plant diseases remain limited. Thus, understanding the pathogenicity mechanisms of *V*. *mali* is crucially important to develop more effective disease management strategies.

Similar to other necrotrophic fungi, *V*. *mali* kills host cells by producing toxins and secreting cell wall‐degrading enzymes (CWDEs), which are considered the major pathogenicity factors (Chen et al., 2012; Wang et al., 2014; Xu et al., 2016). A whole‐genome analysis has suggested that *V*. *mali* has a large number of pathogenicity‐related genes involved in plant cell wall degradation and biosynthesis of secondary metabolites (Feng et al., 2020; Yin et al., 2015). Previous studies have reported that genes encoding the CWDEs play important roles during the pathogenicity of *V*. *mali*. For instance, pectinase secreted by *V*. *mali* causes apple bark tissue maceration and is thus essential to pathogen virulence (Xu et al., 2016). The role in the virulence played by xylanase, involved in the hydrolysis of xylan, was confirmed in plant pathogens such as *Botrytis cinerea* and *Sclerotinia sclerotiorum* (Brito et al., 2006; Yu et al., 2016). One xylanase gene, *VmXyl1*, contributes to the production of pycnidia and is required for the full pathogenicity of *V*. *mali* (Yu et al., 2018). Inactivation of feruloyl esterases in *V*. *mali* leads to reduced virulence (Xu et al., 2018). *V*. *mali* can produce several kinds of nonspecific toxic compounds, both in vivo and in vitro, that are associated with the pathogenicity (Natsume et al., 1982; Wang et al., 2014). However, the genes involved in the production of those toxins remain unknown.

Among the five toxic compounds produced by *V*. *mali*, protocatechuic acid exhibits the greatest phytotoxic activity (Wang et al., 2014). According to the hypothesized toxin production pathway in *V*. *mali*, 4‐hydroxybenzoate is hydroxylated and then converted to protocatechuic acid (Natsume et al., 1982). Previous studies in bacteria suggest that 4‐hydroxybenzoate is predominantly hydroxylated at the third position by 4‐hydroxybenzoate 3‐hydroxylase to yield 3,4‐dihydroxybenzoic acid (protocatechuic acid) (Huang et al., 2008). In *Xanthomonas campestris*, 4‐hydroxybenzoate is hydroxylated by 4‐hydroxybenzoate 3‐hydroxylase for conversion to protocatechuic acid, which can be further metabolized into other products. The 4‐hydroxybenzoate degradation pathway in *X*. *camperstris* is required for its full pathogenicity (Wang et al., 2015). In *Pseudomonas* sp. and *Alcaligenes* sp., 4‐hydroxybenzoate is also degraded by 4‐hydroxybenzoate 3‐hydroxylase via the protocatechuic acid route (Deveryshetty et al., 2007).

The aim of the present study was to identify those hydroxybenzoate hydroxylase (*Hbh*) genes that convert 4‐hydroxybenzoate to protocatechuic acid, and to evaluate their potential functions during infection of apple trees by *V*. *mali*. The published genomic sequence predicted 10 candidate genes that encode hydroxybenzoate hydroxylase (Yin et al., 2015). Transcriptome profiling showed that four of the 10 genes were upregulated during *V*. *mali* infection (*VmHbh1*, *VmHbh2*, *VmHbh3*, and *VmHbh4*), indicating that these four genes may play important roles in fungal virulence. In this study, we obtained the purified fusion protein of VmHbh1 and constructed gene deletion mutants to evaluate their roles in the production of protocatechuic acid and pathogenicity of *V*. *mali*. Of the four genes, two (*VmHbh1* and *VmHbh4*) were required for the full virulence of *V*. *mali* in apple trees.

## 2 RESULTS

### 2.1 Sequence identification and analysis of *V. mali Hbh* genes

**FIGURE 1:**
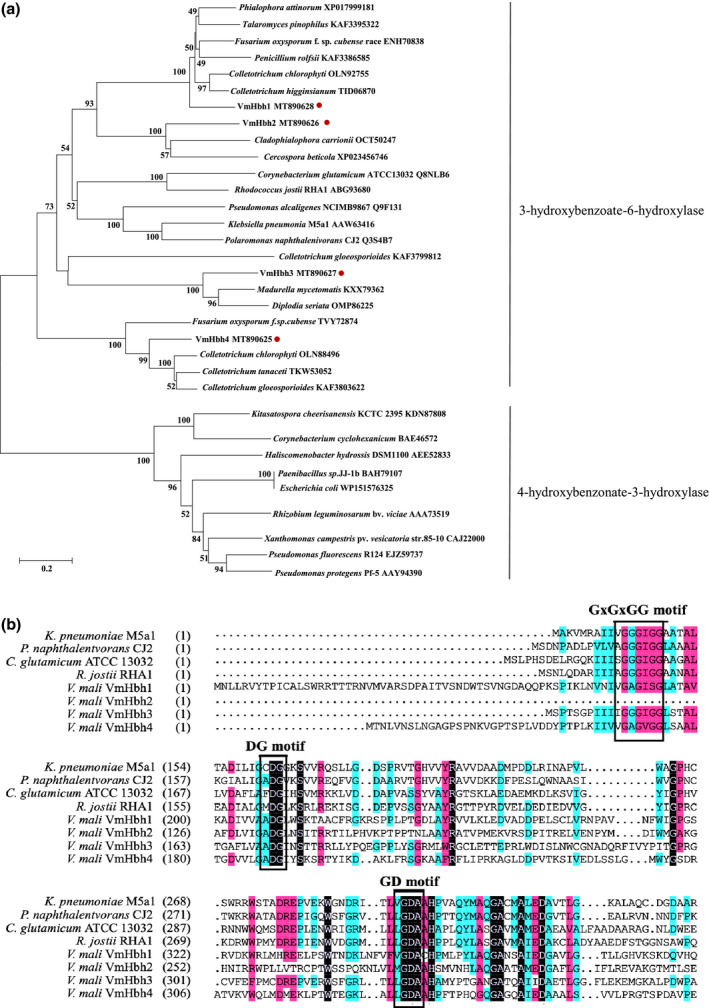
Phylogenetic tree and alignment of the amino acid sequences of hydroxybenzoate hydroxylase (Hbh). (a) Phylogenetic analysis of 3‐hydroxybenzoate 6‐hydroxylase and 4‐hydroxybenzoate 3‐hydroxylase. The phylogram was generated by the maximum‐likelihood algorithm as implemented in MEGA 7. Numbers beside each node indicate bootstrap values as a percentage of 1,000 bootstraps. *Phialophora attinorum* XP017999181, *Talaromyces pinophilus* KAF3395322, *Fusarium oxysporum* f. sp. *cubense* race ENH70838, *Penicillium rolfsii* KAF3386585, *Colletotrichum chlorophyti* OLN92755, *Colletotrichum* *higginsianum* TID06870, *Cladophialophora carrionii* OCT50247, *Cercospora beticola* XP023456746, *Corynebacterium glutamicum* ATCC13032 Q8NLB6, *Rhodococcus jostii* ABG93680, *Pseudomonas alcaligenes* Q9F131, *Klebsiella pneumonia* AAW63416, *Polaromonas naphthalenivorans* Q3S4B7, *Colletotrichum* *gloeosporioides* KAF3799812, *Madurella mycetomatis* KXX79362, *Diplodia seriata* OMP86225, *F*. *oxysporum* f. sp. *cubense* TVY72874, *Colletotrichum* *chlorophyti* OLN88496, *Colletotrichum* *tanaceti* TKW53052, *C*. *gloeosporioides* KAF3803622, *Kitasatospora cheerisanensis* KCTC 2395 KDN87808, *Corynebacterium cyclohexanicum* BAE46572, *Haliscomenobacter hydrossis* AEE52833, *Paenibacillus* sp. BAH79107, *Rhizobium leguminosarum* bv. *viciae* AAA73519, *Xanthomonas campestris* pv. *vesicatoria* CAJ22000. *Pseudomonas fluorescens* EJZ59737, *Pseudomonas protegens* AAY94390. (b) Multiple sequence alignment of the four Hbhs from *Valsa mali* and known 3‐hydroxybenzoate 6‐hydroxylase. Excerpts of sequences were used for multiple sequence alignment, and the aligned sequences are from the phylogenetic tree. Black rectangles indicate conserved motifs of 3‐hydroxybenzoate 6‐hydroxylase

Four candidate *Hbh* genes were amplified by PCR using cDNA of *V*. *mali* as a template and were confirmed by sequencing. The candidate Hbhs all have a monooxygenase flavin‐adenine dinucleotide (FAD)‐binding domain that belongs to the family of group A flavoprotein monooxygenases, which includes 4‐hydroxybenzoate 3‐hydroxylases and 3‐hydroxybenzoate 6‐hydroxylase (Montersino et al., 2011, 2017). A phylogenetic tree was constructed with the characterized proteins of 3‐hydroxybenzoate 6‐hydroxylases and 4‐hydroxybenzoate 3‐hydroxylases from other strains, and confirmed that the four *VmHbh*s were all closely related to the 3‐hydroxybenzoate 6‐hydroxylases (Figure 1a). The sequence alignment of VmHbhs and four characterized 3‐hydroxybenzoate 6‐hydroxylases are given in Figure 1b. VmHbh1, VmHbh3, VmHbh4, and the four reference sequences all have the three flavin‐binding motifs, such as GxGxGG, DG, and GD, whilst VmHbh2 has the two conserved motifs DG and GD (Eppink et al., 1997).

### 2.2 Conversion of 4‐hydroxybenzoate to 3,4‐dihydroxybenzoic acid by VmHbh1

**FIGURE 2:**
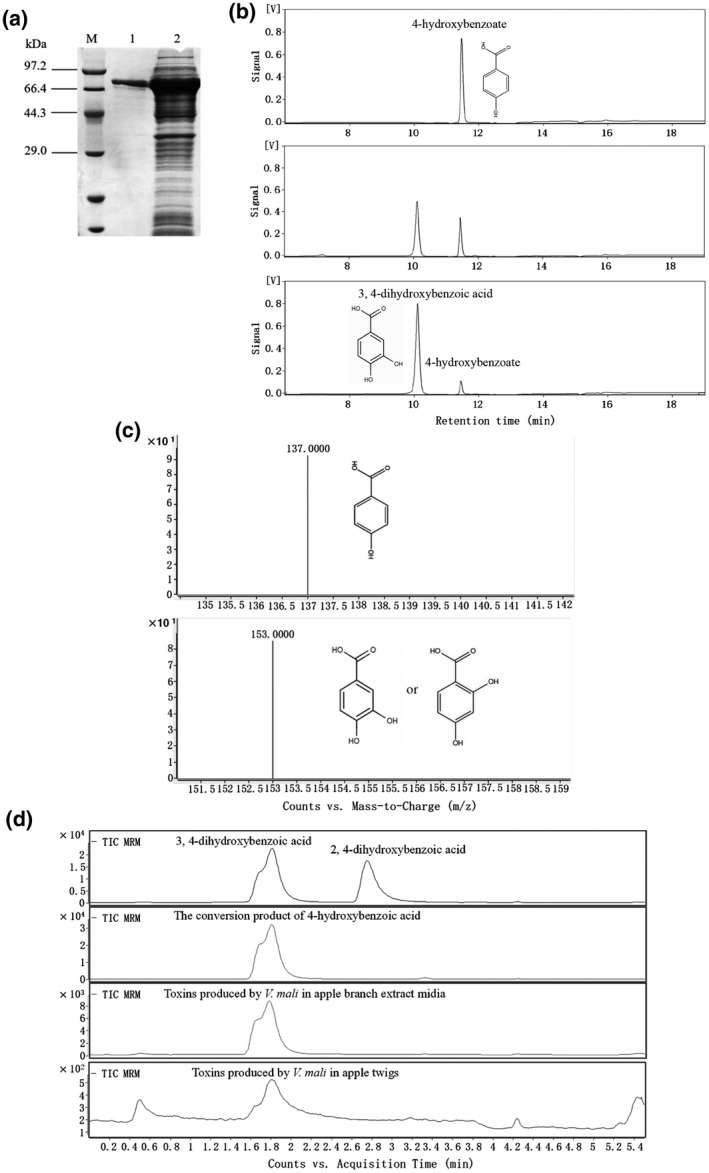
Purification and functional verification of the VmHbh1 recombinant protein. (a) Detection and purification of the VmHbh1 recombinant protein by sodium dodecyl sulphate polyacrylamide gel electrophoresis. Lane M, protein molecular weight marker (low); lane 1, the purified recombinant protein; lane 2, the supernatant of the induced cells at 15 ℃. (b) Enzymatic activity of the VmHbh1 recombinant protein, showing the conversion of 4‐hydroxybenzoate into 3,4‐dihydroxybenzoic acid (protocatechuic acid) as determined by high‐performance liquid chromatography. (c) The substrate and conversion product identified by liquid chromatography‐mass spectrometry. (d) The extracted ion chromatograms of the conversion product of 4‐hydroxybenzoate, and toxins produced by *Valsa mali* in apple branch extract medium and apple twigs

The purified VmHbh1 protein was obtained using a prokaryotic expression technique (Figure 2a). High‐performance liquid chromatography (HPLC) analysis showed that 4‐hydroxybenzoate decreased with the corresponding increase in protocatechuic acid (Figure 2b). Further analysis based on the liquid chromatography‐mass spectrometry (LC‐MS) confirmed that 4‐hydroxybenzoate and protocatechuic acid are the substrate and product, respectively (Figure 2c,d). During the conversion of 4‐hydroxybenzoate by VmHbh1, 2,4‐dihydroxybenzoic acid, which has the same molecular weight as protocatechuic acid, was not detected. Similarly, 2,4‐dihydroxybenzoic acid was not detected in *V*. *mali* culture on apple branch medium nor in *V*. *mali* ‐infected twigs (Figure 2d). These results suggest that *VmHbh* encode *V*. *mali* Hbh, which converts 4‐hydroxybenzoate to protocatechuic acid.

### 2.3 Transcription levels of *VmHbh* genes

**FIGURE 3:**
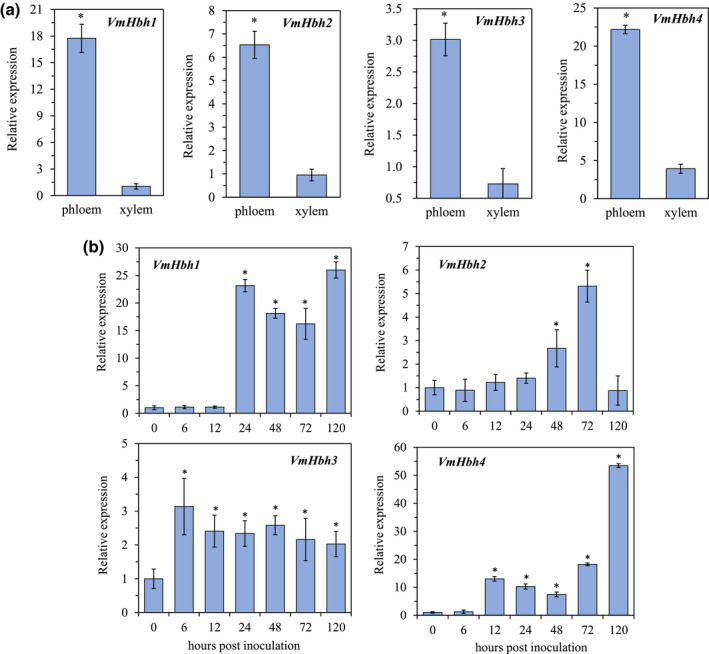
Transcript levels of *VmHbh*s at different conditions and time points postinoculation of apple tissues or twigs with *Valsa mali* as determined by quantitative reverse transcription PCR. The transcript level of *V*. *mali EF1‐a* was used as an endogenous control, and the transcript level of each *VmHbh* gene in the mycelia grown on potato dextrose agar was normalized to 1. The means and standard deviation of the relative expression levels were calculated from three independent biological replicates. (a) Relative expression levels of *VmHbh*s in apple tissues at 72 hr postinoculation (hpi). Asterisks represent significant differences (*p* < 0.05) in transcript levels in the apple phloem and xylem tissues. (b) Relative expression levels of *VmHbh*s at 0, 6, 12, 24, 48, 72, and 120 hpi of apple twigs. Asterisks represent significant differences (*p* < 0.05) in transcript levels as compared to that at 0 hpi

Infected apple phloem and xylem tissues were sampled at 72 hr postinoculation (hpi) to compare the transcript levels of the four *VmHbh*s in the host tissues with those of mycelia grown on potato dextrose agar (PDA). The quantitative reverse transcription‐polymerase chain reaction (RT‐qPCR) analysis showed that the transcript levels of the four *VmHbh*s were significantly upregulated in the apple phloem compared to their respective expression levels in the apple xylem (Figure 3a). This was particularly true for *VmHbh1*: the transcription level in apple phloem was 17.1‐fold higher than that in apple xylem.

The transcript levels of *VmHbh*s during infection of apple twigs by *V*. *mali* were determined for infected apple phloem tissues sampled at seven time points (0, 6, 12, 24, 48, 72, and 120 hpi) as well as for mycelia grown on PDA (Figure 3b). The transcript level of *VmHbh1* was upregulated from 24 to 120 hpi with 16.2‐ to 26.0‐fold increases. Similarly, *VmHbh4* was upregulated from 12 to 120 hpi with 7.5‐ to 53.4‐fold increases. For *VmHbh2*, upregulation was observed only at 48 and 72 hpi with 2.7‐ and 5.3‐fold increases, respectively. The transcript level of *VmHbh3* was upregulated from 6 to 120 hpi with 2.0‐ to 3.1‐fold increases. Overall, the high induction of *VmHbh*s during infection suggested their potential role in the pathogenicity of *V*. *mali*.

### 2.4 *VmHbh*s are not required for growth and pycnidia formation of *V. mali*

For functional analysis of the four *VmHbh*s in *V*. *mali*, we knocked out each of the four genes by the polyethylene glycol (PEG)‐mediated protoplast transformation method. The mutants were initially examined via PCR assays, and then were confirmed by Southern blotting (Figure S2). For the complementation of each *VmHbh* deletion mutant, a complementary construct was generated and transformed into the corresponding mutants. All the complementation transformants were selected using geneticin G‐418 and confirmed by PCR assays with primer pairs ID‐F/ID‐R (Table S1). In addition, RT‐PCR analysis was performed to confirm that the target *VmHbh* was knocked out in the gene deletion mutants, and the complementation strains contained their corresponding target genes (Figure S4).

**FIGURE 4:**
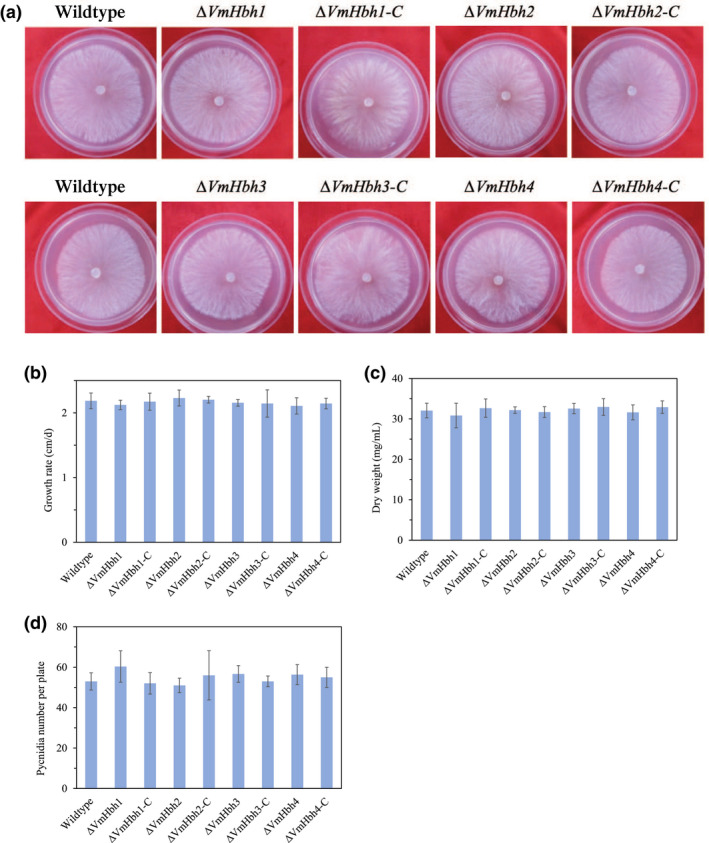
Colony morphology and growth of the wildtype strain and *VmHbh* deletion mutants. (a) Colony phenotypes of different strains growing on potato dextrose agar (PDA) at 25 ℃ in the dark for 2 days. (b) Mycelial growth rate of different strains on PDA at 25 ℃ for 3 days. (c) Mycelium weight of different strains in potato dextrose broth for 7 days with 150 rpm at 25 ℃. (d) Number of pycnidia produced in 7.5 cm diameter Petri plate. Bars indicate standard deviation of means of four replicates

Colony morphology, growth rate, dry mycelia weight, and pycnidia formation of the *VmHbh* deletion mutants and the wildtype strain were measured to determine whether any *VmHbh* played a role in *V*. *mali* development. The *VmHbh* deletion mutants did not differ statistically from the wildtype strains in colony morphology (Figure 4a), growth rate (Figure 4b), and dry mycelia weight after 7 days' culturing in potato dextrose broth (PDB) (Figure 4c). All strains were able to form pycnidia and did not differ in the number of pycnidia produced (Figure 4d). These results indicated that *VmHbh*s did not affect the vegetative growth or pycnidia formation of *V*. *mali*.

### 2.5 Deletion of *VmHbh1* or *VmHbh4* reduce *V. mali* virulence

**FIGURE 5:**
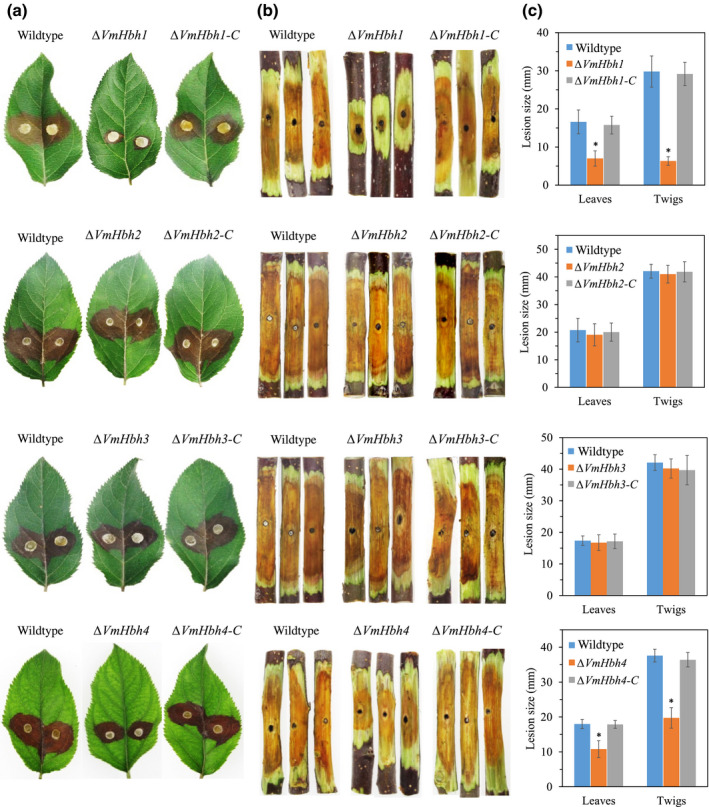
Pathogenicity assays of the wildtype strain, *VmHbh* deletion, and complementation mutants on apple leaves and twigs of *Malus domestica* ‘Fuji’. (a) Infected phenotypes of apple leaves by different strains at 3 days postinoculation (dpi). (b) Infected phenotypes of apple twigs by different strains at 5 dpi. (c) Lesion sizes caused by different strains on apple leaves at 3 dpi and apple twigs at 5 dpi. The mean lesion length was calculated from nine apple leaves and 15 apple twigs. Bars represent the standard deviation. Asterisks indicate significant differences with the wildtype strain (*p* < 0.05)

Pathogenicity assays on the detached apple leaves and twigs were conducted to investigate whether the four *VmHbh* genes play roles in disease development. Pathogenicity was measured in terms of the lesion size following inoculation (Figure 5). Deletion of *VmHbh1* led to at least 65% reduction in lesion sizes on the apple leaves and twigs compared to the wildtype strain. Similarly, the Δ*VmHhb4* mutant also reduced the lesion size on apple leaves and twigs, but to a lesser extent (about 40%), compared to Δ*VmHhb1*. In contrast, the Δ*VmHhb2* and Δ*VmHhb3* mutants did not differ from the wildtype strain in lesion size. To confirm that the reduced pathogenicity of the mutants was caused by the deletion of *VmHbh*s, each complementary construct was generated and transformed into their respective deletion mutants. The previously observed phenotype was rescued in the complementation strains. These results suggested that *VmHhb1* and *VmHhb4* are essential for the pathogenicity of *V*. *mali* in apple leaves and twigs.

### 2.6 Deletion of *VmHbh1* and *VmHbh4* reduce toxin production by *V. mali*

**FIGURE 6:**
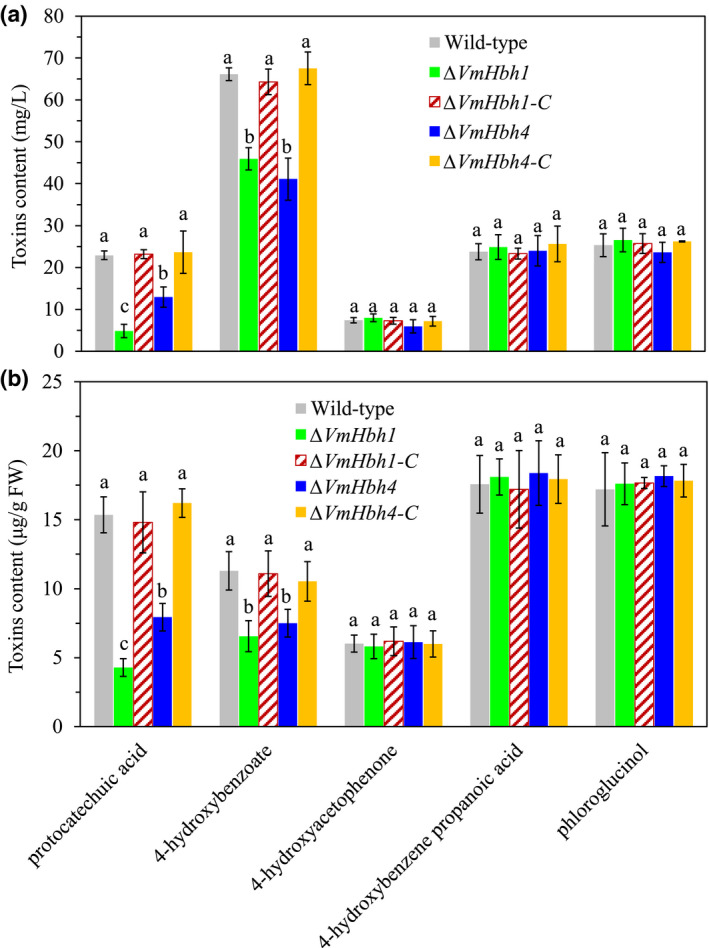
Effects of *VmHbh1* and *VmHbh4* deletion on the toxin levels produced by *Valsa mali*. (a) Toxin levels were determined in the apple branch extract medium for different strains after 7 days of growth. (b) Toxin levels in the apple twigs inoculated with different strains at 7 days postinoculation were analysed with similar amounts of samples. Bars indicate standard deviations of the mean of four replicates. Different letters represent the statistically significant difference (*p* < 0.05) within the same panel

To identify possible mechanisms underlying the reduced pathogenicity of the Δ*VmHhb1* and Δ*VmHhb4* mutants, toxins in the apple branch extract medium under the exposure of the wildtype and mutant strains were quantified. All strains produced five types of toxins, but the Δ*VmHhb1* mutant produced 79% and 31% less protocatechuic acid and 4‐hydroxybenzoate, respectively, in the apple branch extract medium (Figure 6a). Similarly, the Δ*VmHhb4* mutant produced less protocatechuic acid (43% less) and 4‐hydroxybenzoate (38% less) compared to the wildtype strain. Toxins during fungal infection of apple twigs with the wildtype and the deletion strains were also quantified (Figure 6b). The Δ*VmHhb1* and Δ*VmHhb4* mutants had 72% and 48% reductions in protocatechuic acid, respectively; the corresponding values were 42% and 34% for 4‐hydroxybenzoate. There were no significant differences in the quantities of the other three toxic compounds between the wildtype and mutant strains. When *VmHhb1* or *VmHhb4* was reintroduced into the gene deletion mutants, the phenotype was rescued. Neither the Δ*VmHhb2* nor Δ*VmHhb3* mutants differed from the wildtype strain in the toxin levels (data not shown).

### 2.7 Redundancies of *VmHbh*s in the virulence of *V. mali*

**FIGURE 7:**
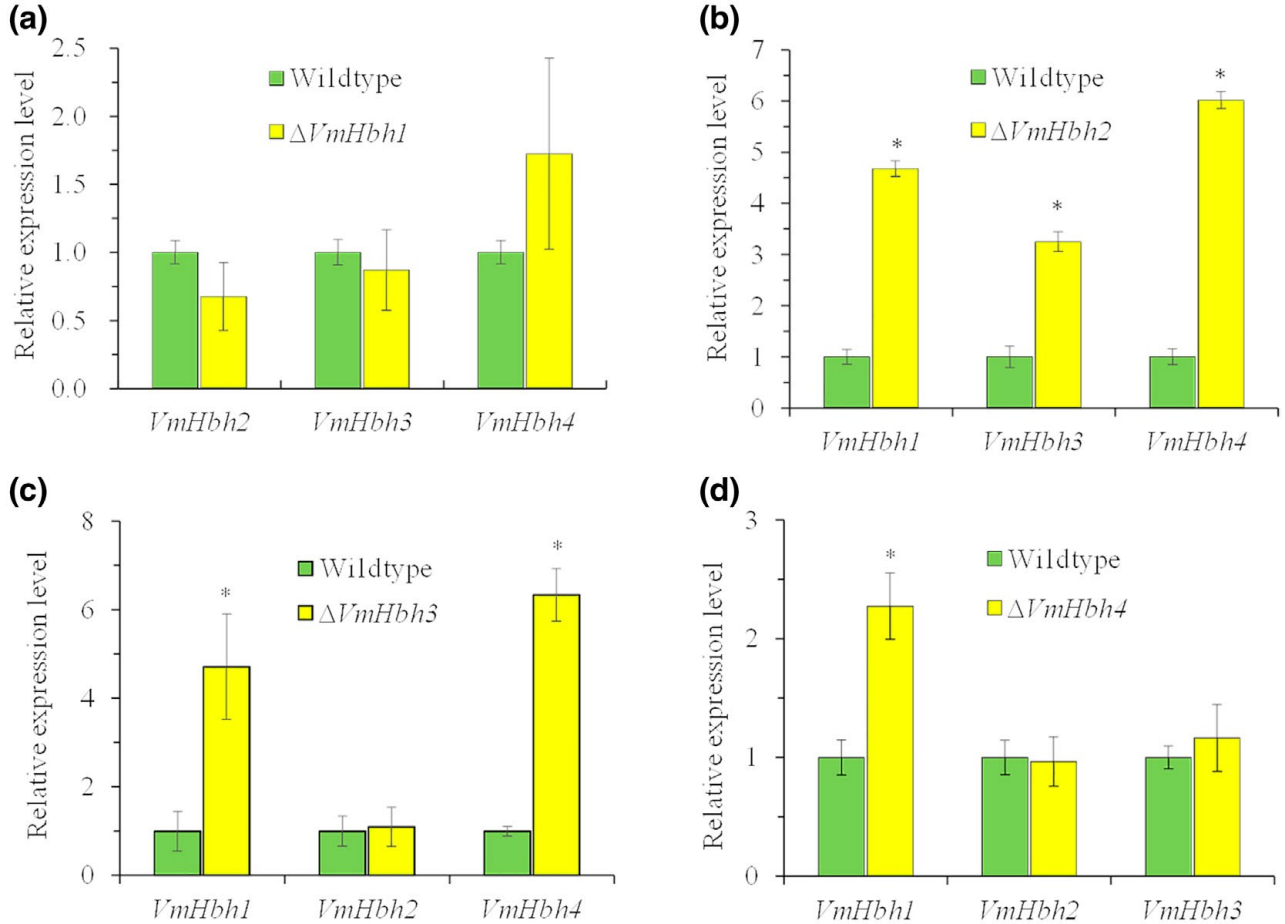
Transcript levels of *VmHbh*s in the wildtype strain and each *VmHbh* gene deletion mutant at 48 hr postinoculation (hpi). (a) Relative expression level of *VmHbh2*, *VmHbh3*, and *VmHbh4* in the ∆*VmHbh1* mutant. (b) Relative expression level of *VmHbh1*, *VmHbh3*, and *VmHbh4* in the ∆*VmHbh2* mutant. (c) Relative expression level of *VmHbh1*, *VmHbh2*, and *VmHbh4* in the ∆*VmHbh3* mutant. (d) Relative expression level of *VmHbh1*, *VmHbh2*, and *VmHbh3* in the ∆*VmHbh4* mutant. The transcript levels of *VmHbhs* in the wildtype strain at 48 hpi were set to 1, and *EF1‐a* was used as the housekeeping gene. The means and standard deviation of the relative expression levels were calculated from three independent biological replicates. Asterisks represent a significant difference in transcript levels (*p* < 0.05)

The RT‐qPCR analysis showed that, when grown on PDA, the transcript levels of *VmHbh*s did not differ between the deletion mutants and the wildtype strain (Figure S5). During infection, the transcript levels of *VmHhb2*, *VmHhb3*, and *VmHhb4* remained at a similar level in the Δ*VmHhb1* mutant relative to that of the wildtype strain (Figure 7a). However, *VmHhb1*, *VmHhb3*, and *VmHhb4* were significantly upregulated (with 4.7‐, 3.2‐, and 6.0‐fold increases, respectively) in the Δ*VmHhb2* mutant relative to the wildtype strain (Figure 7b). Similarly, *VmHhb1* and *VmHhb4* were upregulated in the Δ*VmHhb3* mutant (Figure 7c) as well as the transcript level of *VmHbh1* in the Δ*VmHhb4* mutant (Figure 7d). These data indicate that a deficiency in *VmHbh2*, *VmHbh3*, or *VmHbh4* could stimulate the upregulation of the other genes to different degrees.

## 3 DISCUSSION

Protocatechuic acid, 4‐hydroxybenzoate, 4‐hydroxyacetophenone, 4‐hydrobenzene propanoic acid, and phloroglucinol produced by *V*. *mali* have been described as phytotoxins that can cause necrosis in plants (Natsume et al., 1982; Wang et al., 2014). In *V*. *mali*, strains producing more toxins are more virulent than strains producing fewer toxins (Li et al., 2014; Wang et al., 2014). The five studied toxic compounds are believed to be the degradation products of the phloridzin, but little is known about functional genes that are involved in the phloridzin degradation process. Among these toxic compounds, protocatechuic acid is the most phytotoxic and the last toxic compound in the production pathway of these toxins. Previous studies in bacteria have shown that 4‐hydroxybenzoate is hydroxylated by 4‐hydroxybenzoate 3‐hydroxylase to yield protocatechuic acid (Deveryshetty et al., 2007; Donoso et al., 2011; Romero‐Silva et al., 2013). In the present study, we identified four *VmHbh* genes and demonstrated the role of hydroxybenzoate hydroxylase in the production of toxins and virulence of *V*. *mali* in apple leaves and twigs.

Interestingly, amino acid sequence analysis suggested that these four hydroxybenzoate hydroxylases from *V*. *mali* are more closely related to the 3‐hydroxybenzoate 6‐hydroxylases than to the 4‐hydroxybenzoate 3‐hydroxylases. Moreover, these four VmHbhs have similar conserved motifs to those of 3‐hydroxybenzoate 6‐hydroxylases, namely GxGxGG, DG, and GD (Chen et al., 2018). Previous studies demonstrated that 3‐hydroxybenzoate 6‐hydroxylases can convert 3‐hydroxybenzoate to 2,5‐dihydroxybenzoic acid and show activity when salicylate or 4‐hydroxybenzoate is used as the substrate (Gao et al., 2005; Montersino & van Berkel, 2012). By sequence analysis, we found few functional annotations of 4‐hydroxybenzoate 3‐hydroxylases in fungi in the NCBI database. Until now, the functional analysis and crystal structure of 4‐hydroxybenzoate 3‐hydroxylases and 3‐hydroxybenzoate 6‐hydroxylase are all from bacteria or yeast (van Berkel et al., 1994; Hiromoto et al., 2006; Holesova et al., 2011; Montersino et al., 2013; Schreuder et al., 1988). This is the first report that hydroxybenzoate hydroxylase (VmHbh) from *V*. *mali* can convert 4‐hydroxybenzoate to protocatechuic acid but does not have enzymatic activities when the substrate is 3‐hydroxybenzoate (data not shown). Therefore, the present results indicate that VmHbh1 performs the function of 4‐hydroxybenzoate 3‐hydroxylase in *V*. *mali*.

During infection by *V*. *mali*, all four *VmHbh*s genes showed high transcript levels, especially *VmHbh1* and *VmHbh4*, indicating that these genes may play a role in pathogenicity toward apples. Furthermore, RT‐qPCR analysis also demonstrated that the greatest upregulation of their expression occurred at different time points: *VmHbh1*, *VmHbh3*, and *VmHbh4* were upregulated at 6–24 hpi, whereas *VmHbh2* was upregulated at 48 hpi. The differential temporal dynamics in the transcripts may reflect that these genes play important roles at different time points during infection. In addition, the four *VmHbh* genes showed significantly higher expression levels in phloem than in xylem tissues. Previous studies indicated that *V*. *mali* can grow rapidly and survive for a long time in xylem, but it could not develop disease symptoms until the pathogen reached the phloem (Chen et al., 2016; Wang et al., 2018). Thus, the transcript levels of the *VmHbh* genes may reflect the pathogenicity level of *V*. *mali* in apple tissues.

In this study, we generated gene deletion mutants of all four *VmHbh* genes and demonstrated that *VmHbh*s are not necessary for mycelial growth and pycnidia formation in *V*. *mali*. In contrast, the pathogenicity‐related gene *BcKMO* in *B. cinerea*, encoding kynurenine 3‐monooyxgenase (KMO) with a monooxygenase FAD‐binding domain, is known to be important for fungal development in *B*. *cinerea*, and its mutant does not produce conidia or sclerotia, and has a lower rate of growth (Zhang et al., 2018). These data indicate that the contribution of monooxygenases to fungal growth and development may depend on the pathogen species.

*VmHbh2* or *VmHbh3* did not influence the virulence of *V*. *mali*, but the pathogenicity of the deletion mutants of *VmHbh1* or *VmHbh4* showed large reductions compared to the wildtype strain. This may imply functional redundancies of the genes in *V*. *mali* pathogenicity. Such a functional redundancy also exists in other gene families of *V*. *mali*, for instance the abundant effectors and CWDEs that act as virulence factors (Xu et al., 2018; Zhang et al., 2019). Moreover, the RT‐qPCR analysis of each gene, with the exception of *VmHbh1*, showed a complementary effect in the deletion mutants. When *VmHbh1* was knocked out, the expression of other three *VmHbh*s was not upregulated, which indicates that these genes are unlikely to complement the function of *VmHbh1*. For the deletion of *VmHbh4*, only *VmHbh1* was upregulated with a lower increase, which may partly complement the function of the deleted *VmHbh4*. However, in the gene deletion mutants of *VmHbh2* and *VmHbh3*, the expression levels of the other three *VmHbh*s were significantly upregulated. These results indicate that the upregulated *VmHbh1* and *VmHbh4* could rescue the function of the four deleted genes, and hence the deletion of *VmHbh2* or *VmHbh3* did not impair the virulence of *V*. *mali*. A similar result has been found previously with regard to Hce2‐containing effectors of *V*. *mali* such as *VmHEP1* and *VmHEP2* (Zhang et al., 2019). The deletion of one *VmHEP* promoted the expression of the other one, meanwhile the deletion of each single *VmHEP* did not lead to a reduction in the virulence. In another study of polygalacturonase (PG), the transcript levels of three genes in the PG family were significantly induced in strains with the double‐deletion of *Vmpg7* and *Vmpg8* (Xu et al., 2016).

An important discovery of our work is the finding that the deletion of *VmHbh1* and *VmHbh4* significantly reduced levels of toxin, which may explain the corresponding decrease in the virulence of *V*. *mali*. This is consistent with the previous results of the *V*. *mali* xylanase gene *VmXyl1*, which contributes significantly to both the total xylanase activity and virulence (Yu et al., 2018). Similarly, the deletion of feruloyl esterase genes decreases the capacity of *V*. *mali* to utilize ethyl ferulate, which is consistent with reduced virulence of the gene deletion mutants (Xu et al., 2018). In contrast, polyketide synthase BcPKS6 and BcPKS9 are key enzymes for the biosynthesis of botcinic acid, an important phytotoxin during infection by *B*. *cinerea*: when one of theses two genes is deleted, the loss of botcinic acid production does not affect the virulence of *B*. *cinerea* in plant tissues (Dalmais et al., 2011). In this study, the fact that deletion of *VmHbh2* or *VmHbh3* did not affect the toxin levels demonstrated redundant functions in toxin production.

In the present study, we demonstrated that VmHbh1 can convert 4‐hydroxybenzoate to protocatechuic acid. *VmHbh*s are required for full pathogenicity of *V*. *mali* against apple trees. The present findings provide a new perspective for the contribution of Hbhs to the growth, development, and virulence of phytopathogenic fungi.

## 4 EXPERIMENTAL PROCEDURES

### 4.1 Strains and culture conditions

The *V*. *mali* wildtype strain LXS080601 was grown on potato dextrose agar (PDA, 200 g potato, 20 g dextrose, 15 g agar per L) at 25 ℃ in the dark. The gene deletion and complemented mutants were cultured on PDA supplemented with 100 mg/ml hygromycin B or geneticin G‐418 (Sigma). *Escherichia coli* strains were grown in lysogeny broth (LB) with appropriate antibiotics at 37 ℃. Colony diameter was measured to calculate the growth rate of different strains on PDA.

### 4.2 Identification of *Hbh* genes in *V. mali*

To identify the four candidate *Hbh* genes in *V*. *mali* genome, BLAST searches with well‐characterized *Hbh* genes were conducted. The conserved domains of the candidate Hbh proteins were confirmed using the BLAST‐based NCBI conserved domain search engine (Marchler‐Bauer et al., 2017). Primer pairs for the cloning of the four candidate *Hbh* genes were synthesized by TsingKe (Beijing, China) (Table S1). Total RNA was extracted from fungal mycelia with the RNAiso Plus Kit, and cDNA was synthesized with the Prime Script RT reagent kit with the gDNA Eraser (TaKaRa) with an oligo (dT)_12–18_ primer. PCRs were performed with PrimerSTAR Max DNA polymerase and the PCR products were cloned to T‐Vector pMD 19 Simple (TaKaRa), according to the manufacturer's instructions.

### 4.3 Sequence alignment and phylogenetic analysis

The amino acid sequences of Hbhs from other microorganisms in this study were obtained from the NCBI GenBank. All the homology searches were carried out on the NCBI BLAST server. These obtained sequences were compared with the sequence from *V*. *mali* (MT890625, MT890626, MT890627, and MT890628). The maximum‐likelihood (ML) method, as implemented in MEGA 7, was used to infer the phylogenetic tree with 1,000 bootstrapping replicates. Multiple sequence alignments of *VmHbh*s and other published *Hbh* genes (*Klebsiella pneumoniae*, AAW63416; *Polaromonas naphthalenivorans*, Q3S4B7; *Corynebacterium glutamicum*, Q8NLB6; *Rhodococcus jostii*, ABG93680) were performed using DNAMAN v. 6.0 with all parameters set at the default values.

### 4.4 Hydroxybenzoate hydroxylase activity assay

To assay the hydroxybenzoate hydroxylase activity of VmHbhs, the cDNA fragments were subcloned into pET‐32a or pMal‐c2× by homologous recombination using the ClonExpress Ⅱ One Step Cloning Kit (Vazyme). The vector constructs were transformed into *E. coli* Rosetta whilst the soluble recombinant proteins of VmHbh1 and VmHbh4 were obtained after induction with 0.5 mM isopropyl β‐d‐thiogalactopyranoside (IPTG) for 16 hr at 15 ℃ (Shi et al., 2015). We used amylose resin (New England Biolabs) or Ni‐NTA spin column (Qiagen) to purify the recombinant proteins.

The hydroxybenzoate hydroxylase activity was measured following Chen et al. (2018) with some modifications. Activity was assayed in 1 ml of the reaction mixture containing 400 µM 4‐hydroxybenzoate, 400 µM NADH, and about 15 µg of purified recombinant protein in 50 mM phosphate buffer (pH 8.0) at 25 ℃. During the conversion, the concentrations of 4‐hydroxybenzoate and protocatechuic acid were determined by HPLC and LC‐MS (1200 series, 1290 Infinity/6460 ultra HPLC‐MS; Agilent Technologies).

### 4.5 Detection of gene expression by RT‐qPCR

Mycelia grown on PDA for 3 days were used to inoculate apple twigs. Apple bark around the lesion margin was sampled at 0, 6, 12, 24, 48, 72, and 120 hpi. For samples at 0 hpi, the bark tissues around inoculation sites containing mycelium plugs were collected (Yu et al., 2018). The RNA was extract from bark tissues using the RNAiso Plus Kit (TaKaRa), and then the cDNA was synthesized. All RT‐qPCR experiments were conducted in a LightCycler 480ⅠⅠ PCR detection system (Roche) with SYBR Master Mix (TaKaRa), following the manufacturer's protocol. The *V*. *mali EF1‐a* gene was used as an endogenous control, and the primers are given in Table S1. The relative expression levels of each gene were calculated using the 2^−ΔΔ^ *^C^* ^t^ method. Data from three biological replicates were used to calculate the means and standard deviation. The whole experiment was repeated twice.

### 4.6 Generation of gene deletion and complementation strains

To obtain the *VmHbh* gene deletion mutants, the PEG‐mediated homologous recombination was performed as described previously (Yu et al., 2018). The gene deletion cassette with three components used the hygromycin B phosphotransferase gene (*HPH*) as a selective marker for the gene deletion (Figure S1). Upstream and downstream fragments of *VmHbh* genes were amplified from genomic DNA of the wildtype strain LXS080601 using the gene‐specific primer pairs Up‐F/Up‐R and Down‐F/Down‐R (Table S1), respectively. The *HPH* gene was amplified with primers HPH‐F and HPH‐R from the vector pBS (Table S1). The gene deletion cassette was generated by double‐joint PCR, which was confirmed by the sequencing. The cassettes were later transformed into the protoplasts of *V*. *mali* LXS080601, and the transformants were screened by culturing on medium with 100 μg/ml hygromycin (Yu et al., 2018). The putative gene deletion mutants were validated by the PCR using four primer pairs (Figure S1 and Table S1). Primers I‐F and I‐R were used to detect the fusion segment; HPH‐P‐F and HPH‐P‐R to detect the resistance gene (*HPH*). The Up‐F and Full‐R were used to confirm if the upstream sequence of the replaced resistance gene was fused at the right position, and Full‐F and Down‐R to confirm whether the downstream sequence of the introduced resistance gene was fused at the right position. The gene deletion was finally verified by Southern hybridization with a DIG DNA Labeling and Detection Kit Ⅱ (Roche), following the instruction manual. Genomic DNA was digested with appropriate restriction enzymes and separated via agarose gel electrophoresis. The *HPH* gene used for the replacement cassette served as probe.

To construct the gene complement vector, the entire *VmHbh* genes and predicted promoter sequences were amplified from genomic DNA using the primer pair CM‐F/CM‐R (Table S1). The PCR products were cloned into pYF11 using yeast gap repair, as described previously (Yu et al., 2014). Fusion constructs were verified by the sequencing analysis, and plasmids were transformed into the corresponding gene deletion mutant using the PEG‐mediated method. The transformants were selected using G‐418 and confirmed by PCR with the corresponding primers (Table S1).

### 4.7 RT‐PCR detection of *VmHbhs* in the wildtype, gene deletion mutants, and complementation mutants

The RNA extraction from cultured mycelia of the wildtype strain, *VmHbh* deletion mutants, and complementation mutants were performed as described previously. The cDNA was synthesized and the primers Full‐F/Full‐R were employed to detect the *VmHbh* genes (Table S1). The PCR conditions were as follows: 30 cycles of 94 ℃ for 30 s, 55 ℃ for 45 s, and 72 ℃ for 60 s; with a final extension at 72 ℃ for 5 min. The housekeeping gene *EF1‐a* was used as a control.

### 4.8 Vegetative growth, pycnidia formation, and pathogenicity assays

Mycelium plugs (5 mm diameter) cut from actively growing colony edges of the wildtype strain, gene deletion, and complemented mutants were transferred to new PDA plates. The plates were then incubated at 25 ℃ before colony shape, colour, and colony diameters were assessed. For the dry weight of mycelia, plugs were inoculated into potato dextrose broth at 150 rpm and 25 ℃ for 7 days.

Pathogenicity assays were performed on the apple leaves and 1‐year‐old twigs (*Malus domestica* ‘Fuji’) were taken from the greenhouse at Qingdao Agricultural University, Qingdao, China. The detached leaves and twigs were sterilized with 75% ethanol and the wounds were made as described by Yu et al. (2018). Mycelium plugs were used to inoculate the wounds. The inoculated leaves and twigs were placed in trays to maintain high humidity at 25 ℃ in the dark. Then, the lesion length was measured and lesion development was photographed at several time points. The assays were repeated three times, with at least 15 leaves and twigs per treatment.

### 4.9 Determination of toxins production

For toxins production in the liquid media, mycelium plugs of the wildtype strain, gene deletion mutants, and complemented mutants were inoculated into apple branch extract medium. After culturing for 7 days under 150 rpm/min and 25 ℃ with a 12‐hr photoperiod, the supernatant was collected. The medium inoculated with PDA was used as a control. For toxin production in apple tissues, detached twigs were inoculated according to the method in section 4.8. After 7 days, the diseased areas and margins of the twigs were collected and crushed into small pieces. Toxins were extracted according to a method described by Wang et al. (2014). The final samples were filtered through a 0.22‐μm pore membrane for the HPLC analysis. Each experiment was repeated two times.

## CONFLICT OF INTEREST

The authors declare that no competing interests exist.

## Supporting information


**FIGURE S1** Schematic view of targeted deletion of *VmHbh*s by the homologous recombination method. The small arrows mark the positions and directions of the primers used for PCR. The *HPH* gene was amplified with the primer pair HPH‐F/HPH‐R. The upstream and downstream flanking sequences were amplified with Up‐F/Up‐R and Down‐F/Down‐R, respectively. The fusion fragment of upstream, *HPH*, and downstream was amplified with the primer pair Nest‐F/Nest‐R. All sequences are given in Table S1

**FIGURE S2** Deletion of *VmHbhs* verified by PCR and Southern blotting analyses. Detection of *VmHbh* deletion mutants by PCR with four primer pairs I‐F/I‐R (a, detection of fusion segment), Up‐F/Full‐R and Full‐F/Down‐R (b, detection of upstream and target gene, detection of downstream and target gene), and HPH‐F/HPH‐R (c, detection of resistance gene). (d) Further confirmation of individual *VmHbh* gene deletion mutants

**FIGURE S3** Confirmation of complementation strains by PCR. The primer pairs *VmHbh*‐ID‐F/R were used to detect the complementary fragments from the genomic DNA of each *VmHbh* gene complementation strain

**FIGURE S4** Detection of *VmHbh*s from the wildtype strain, *VmHbh* deletion mutants, and complementation strains by reverse transcription PCR. The housekeeping gene *EF1‐a* was used as control

**FIGURE S5** Transcript levels of *VmHbh*s in the wildtype strain and each *VmHbh* gene deletion mutant grown on potato dextrose agar for 3 days. (a) Relative expression level of *VmHbh2*, *VmHbh3*, and *VmHbh4* in the *∆VmHbh1* mutant. (b) Relative expression level of *VmHbh1*, *VmHbh3*, and *VmHbh4* in the *∆VmHbh2* mutant. (c) Relative expression level of *VmHbh1*, *VmHbh2*, and *VmHbh4* in the *∆VmHbh3* mutant. (d) Relative expression level of *VmHbh1*, *VmHbh2*, and *VmHbh3* in the *∆VmHbh4* mutant. The transcript levels of *VmHbh*s in the wildtype strain were set to 1 and *EF1‐a* was used as the housekeeping gene. The means and standard deviations of the relative expression levels were calculated from three independent biological replicates

**TABLE S1** Primers used in this study

## Data Availability

The data that support the findings of this study are available from the corresponding author upon reasonable request.
