# Supplementary material for: Hydroxybenzoate hydroxylase genes underlying protocatechuic acid production in Valsa mali are required for full pathogenicity in apple trees
Source: Mol Plant Pathol. 2021 Aug 13;22(11):1370–82. doi: 10.1111/mpp.13119 (PMC8518569; doi:10.1111/mpp.13119)
Supplement: Supplementary file 6 — TABLE S1 Primers used in this study [file MPP-22-1370-s004.docx]

**Supplementary Table**

**Supplementary Table S1. Primers used in this study**

| **Fragments Use** | **Primers** | **Sequence (5'-3')** |  |
| --- | --- | --- | --- |
| Amplification ORF | *VmHbh1*-F | ATGAATCTACTGAGAGTTTATAC | |
|  | *VmHbh1*-R | TTAGAACGGCTCGTCTTTGA | |
|  | *VmHbh2*-F | ATGCTCGAATCCGCGTCCGA | |
|  | *VmHbh2*-R | CAGAGTAGCGATCTGATCTTCAGG | |
|  | *VmHbh3*-F | ATGTCACCCACTTCCGGACC | |
|  | *VmHbh3*-R | CTAATTAAATCGTTTCGCCAGAGAC | |
|  | *VmHbh4*-F | ATGACGAACCTGGTGAATAG | |
|  | *VmHbh4*-R | TTACTCAATCCTCCTCGCA | |
| qRT-PCR | *EF1-α*-F | TGAGTTCGAGGCTGGTATCTCCAA | |
|  | *EF1*-*α*-R | TGTCCATCTTGTTGATGGCGACGA | |
|  | qRT-*VmHbh1*-F | CCAACCTCATTAAGCGTCCAC | |
|  | qRT-*VmHbh1*-R | CCAGCTGCTTTGCTCGTTC | |
|  | qRT-*VmHbh2*-F | TATAGACCTTGCCGCCGCTGA | |
|  | qRT-*VmHbh2*-R | ACCCGATCACCAGAGAGCTTG | |
|  | qRT-*VmHbh3*-F | CGAAATGCGCCGTGATCCCAT | |
|  | qRT-*VmHbh3*-R | ACCACATCCTCTCAGAGCCGCTA | |
|  | qRT-*VmHbh4*-F | CCATCGCGTTTACCTTCACGAG | |
|  | qRT-*VmHbh4*-R | GTTCACGCTAACCACCTTGCT | |
| Prokaryotic expression of  *VmHbhs* | PET-T7-F | TAATACGACTCACTATAGGG | |
|  | PET-T7-R | GGTTATGCTAGTTATTGCTC | |
|  | *VmHbh1*-YH-F | gctgatatcggatccgaattcATGAATCTACTGAGAGTTTATACACCCAT | |
|  | *VmHbh1*-YH-R | ctcgagtgcggccgcaagcttTTAGAACGGCTCGTCTTTGACC | |
|  | *VmHbh2*-YH-F | gctgatatcggatccgaattcATGCTCGAATCCGCGTCCGA | |
|  | *VmHbh2*-YH-R | ctcgagtgcggccgcaagcttTCAGAGTAGCGATCTGATCTTCAGG | |
|  | *VmHbh3*-YH-F | gctgatatcggatccgaattcATGTCACCCACTTCCGGACC | |
|  | *VmHbh3*-YH-R | ctcgagtgcggccgcaagcttCTAATTAAATCGTTTCGCCAGAGA | |
|  | *VmHbh4*-YH-F | gctgatatcggatccgaattcATGACGAACCTGGTGAATAG | |
|  | *VmHbh4*-YH-R | ctcgagtgcggccgcaagcttTTACTCAATCCTCCTCGCA | |
| Deletion of *VmHbhs* | *VmHbh1*-Up-F | CTATTCTGAGGTCTGTGCGTCGGG | |
|  | *VmHbh1*-Up-R | caaaataggcattgatgtgttgacctccGGGTAGAAACCCGGGA | |
|  | *VmHbh2*-Up-F | TGGTGTTCGAAAGATACGAGG | |
|  | *VmHbh2*-Up-R | caaaataggcattgatgtgttgacctccCGCGTATATACAGGACAACGT | |
|  | *VmHbh3*-Up-F | AACAGGATGAACGCGTAAGTA | |
|  | *VmHbh3*-Up-R | caaaataggcattgatgtgttgacctccATACCTAGGCACTCATCTCTT | |
|  | *VmHbh4*-Up-F | GCCACACAAGCAATAGCCACT | |
|  | *VmHbh4*-Up-R | caaaataggcattgatgtgttgacctcc ATGATACCGTTGAGGGAACCT | |
|  | *VmHbh1*-Down-F | ctcgtccgagggcaaaggaatagagtagACACCTGGGCAG | |
|  | *VmHbh1*-Down-R | CTCCCCAACTTCTGGCGGCTG | |
|  | *VmHbh2*-Down-F | ctcgtccgagggcaaaggaatagagtagATTATGCTTGGTTAGTCGGTG | |
|  | *VmHbh2*-Down-R | ATAAGCGATATCCTAAGTGCC | |
|  | *VmHbh3*-Down-F | ctcgtccgagggcaaaggaatagagtagCAGGGATAGAATGCAGTAGTC | |
|  | *VmHbh3*-Down-R | ACATCTAGTACACCTTCCACG | |
|  | *VmHbh4*-Down-F | ctcgtccgagggcaaaggaatagagtag TGACGGGAGGTTGTGGTCATT | |
|  | *VmHbh4*-Down-R | TTCGTCGTTGTGGTCGGTGAT | |
|  | *VmHbh1*-nest-F | TATTCTGAGGTCTGTGCGTC | |
|  | *VmHbh1*-nest-R | CGAGATAGCAAACTTCCCGC | |
|  | *VmHbh2*-nest-F | CACCTATGACAACCCAGGCAT | |
|  | *VmHbh2*-nest-R | CGTGGCCTCTTATCTGATAG | |
|  | *VmHbh3*-nest-F | AGTGCAAAGGAGACATCTGGT | |
|  | *VmHbh3*-nest-R | TATCGAAACGGCACCTCTACA | |
|  | *VmHbh4*-nest-F | CGGGGTAAGTTCGGTCAATAT | |
|  | *VmHbh4*-nest-R | TTTCTTGCCATCTACGACGCT | |
|  | *HPH-*F | GGAGGTCAACACATCAATGCC | |
|  | *HPH-*R | CTACTCTATTCCTTTGCCCTCGG | |
| Complementary of *VmHbhs* | *VmHbh1*-Native-F | actcactatagggcgaattgggtactcaaattggttTCTACCCAATGACGAAGCGG | |
|  | *VmHbh1*-Native-R | caccaccccggtgaacagctcctcgcccttgctcacGAACGGCTCGTCTTTGACC | |
|  | *VmHbh2*-Native-F | actcactatagggcgaattgggtactcaaattggttGACTGACGGATTATTGAG | |
|  | *VmHbh2*-Native-R | caccaccccggtgaacagctcctcgcccttgctcac GTGGAAGAACGAGGTTGCAAT | |
|  | *VmHbh3*-Native-F | actcactatagggcgaattgggtactcaaattggttGCTACATGGTCACATACGC | |
|  | *VmHbh3*-Native-R | caccaccccggtgaacagctcctcgcccttgctcac GTCAAGCTCAAGGGTAGGTCA | |
|  | *VmHbh4*-Native-F | actcactatagggcgaattgggtactcaaattggttTGGAACAACGCCGAGTACACC | |
|  | *VmHbh4*-Native-R | caccaccccggtgaacagctcctcgcccttgctcac CTCAATCCTCCTCGCAGCGCTA | |
|  | *VmHbh1*-ID-F | TCAGACAGGTAAGCACTCAG | |
|  | *VmHbh1*-ID-R | CGTGCTGCTTCATGTGGTCG | |
|  | *VmHbh2*-ID-F | CTACCGTCAAAGACAAGT | |
|  | *VmHbh2*-ID-R | CGTGCTGCTTCATGTGGTCG | |
|  | *VmHbh3*-ID-F | GCGGTTGAACATCCGCAGC | |
|  | *VmHbh3*-ID-R | CGTGCTGCTTCATGTGGTCG | |
|  | *VmHbh4*-ID-F | GTTCGTCGACCTGAGCCTCAC | |
|  | *VmHbh4*-ID-R | CGTGCTGCTTCATGTGGTCG | |
