# Supplementary material for: Hydroxybenzoate hydroxylase genes underlying protocatechuic acid production in Valsa mali are required for full pathogenicity in apple trees
Source: Mol Plant Pathol. 2021 Aug 13;22(11):1370–82. doi: 10.1111/mpp.13119 (PMC8518569; doi:10.1111/mpp.13119)
Supplement: Supplementary file 2 [file MPP-22-1370-s001.docx]

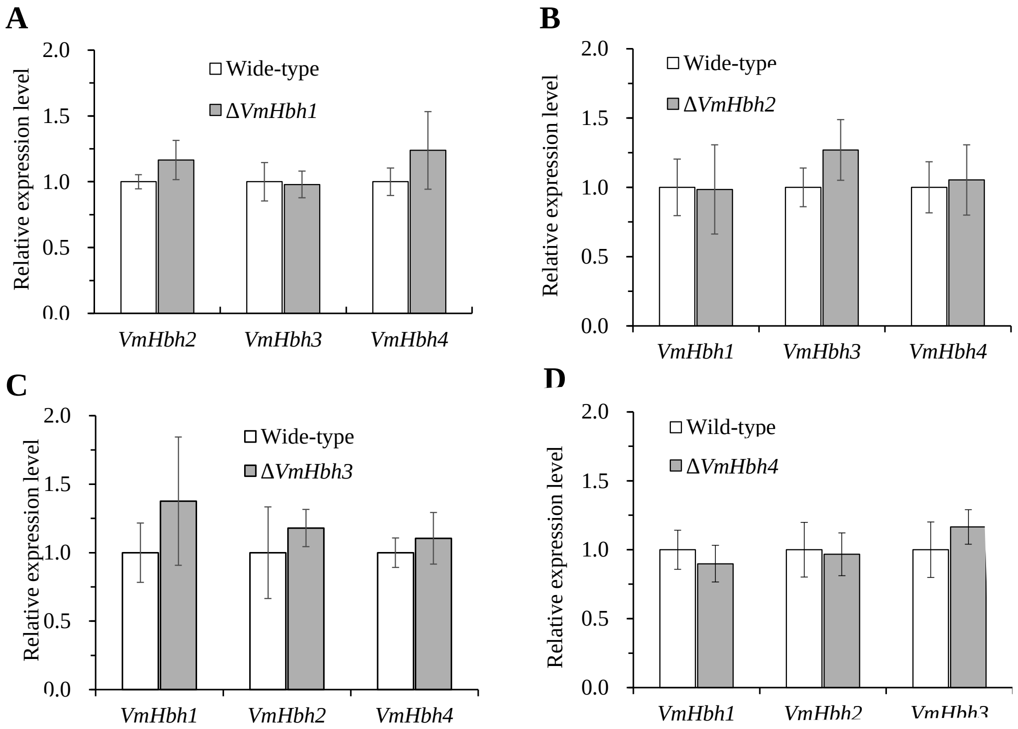

A
Relative expression level
2.0
1.5
1.0
0.5
0.0
Wide-type
ΔVmHbh1
VmHbh2
VmHbh3
VmHbh4
B
Relative expression level
Wide-type
ΔVmHbh2
VmHbh1
VmHbh3
VmHbh4
C
Relative expression level
Wide-type
ΔVmHbh3
VmHbh1
VmHbh2
VmHbh4
D
Relative expression level
Wild-type
ΔVmHbh4
VmHbh1
VmHbh2
VmHbh3
