# Supplementary material for: Hydroxybenzoate hydroxylase genes underlying protocatechuic acid production in Valsa mali are required for full pathogenicity in apple trees
Source: Mol Plant Pathol. 2021 Aug 13;22(11):1370–82. doi: 10.1111/mpp.13119 (PMC8518569; doi:10.1111/mpp.13119)
Supplement: Supplementary file 3 [file MPP-22-1370-s002.docx]

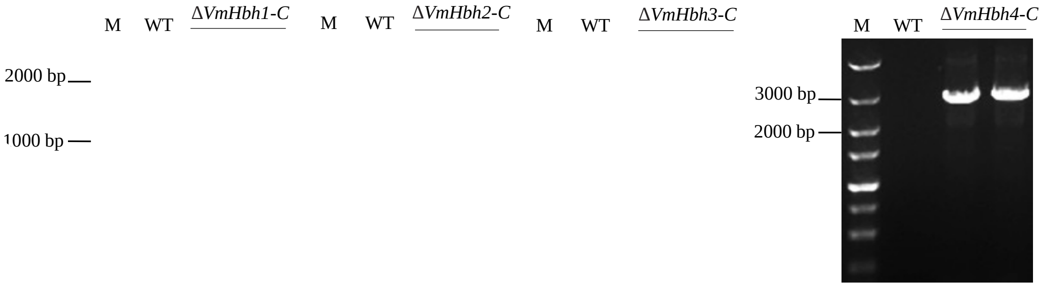
M
WT
ΔVmHbh1-C
M
WT
ΔVmHbh2-C
M
WT
ΔVmHbh3-C
M
WT
ΔVmHbh4-C
2000 bp
1000 bp
3000 bp
2000 bp
