# Supplementary material for: Hydroxybenzoate hydroxylase genes underlying protocatechuic acid production in Valsa mali are required for full pathogenicity in apple trees
Source: Mol Plant Pathol. 2021 Aug 13;22(11):1370–82. doi: 10.1111/mpp.13119 (PMC8518569; doi:10.1111/mpp.13119)
Supplement: Supplementary file 4 [file MPP-22-1370-s003.docx]

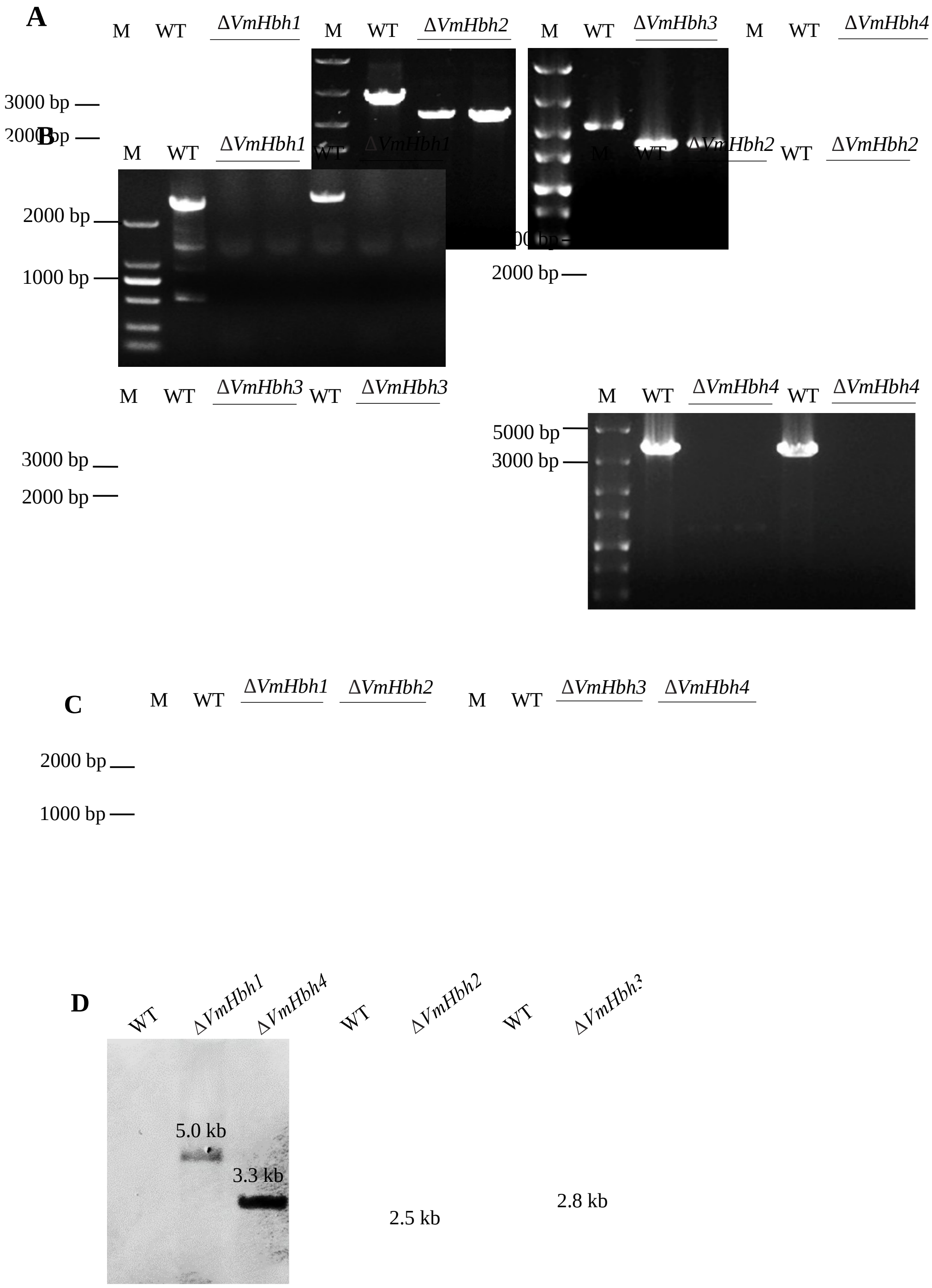

A
M WT ΔVmHbh1 M WT ΔVmHbh2 M WT ΔVmHbh3 M WT ΔVmHbh4
3000 bp
2000 bp
B
M WT ΔVmHbh1 WT ΔVmHbh1 M WT ΔVmHbh2 WT ΔVmHbh2
2000 bp
1000 bp
2000 bp
M WT ΔVmHbh3 WT ΔVmHbh3 M WT ΔVmHbh4 WT ΔVmHbh4
3000 bp
2000 bp
5000 bp
3000 bp
C
M WT ΔVmHbh1 ΔVmHbh2 M WT ΔVmHbh3 ΔVmHbh4
2000 bp
1000 bp
D
WT ΔVmHbh1 ΔVmHbh4 WT ΔVmHbh2 WT ΔVmHbh3
5.0 kb
3.3 kb
2.5 kb
2.8 kb
