# Supplementary material for: Hydroxybenzoate hydroxylase genes underlying protocatechuic acid production in Valsa mali are required for full pathogenicity in apple trees
Source: Mol Plant Pathol. 2021 Aug 13;22(11):1370–82. doi: 10.1111/mpp.13119 (PMC8518569; doi:10.1111/mpp.13119)
Supplement: Supplementary file 5 [file MPP-22-1370-s005.docx]

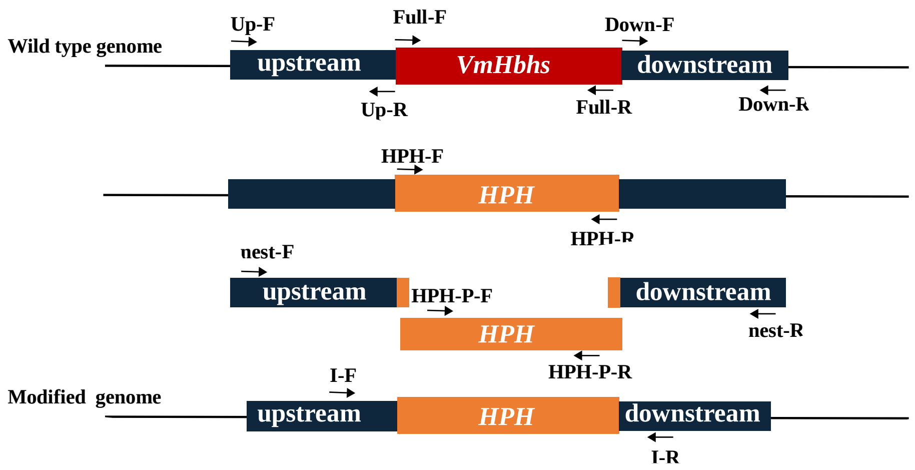

Wild type genome
Up-F
Full-F
Down-F
upstream
VmHbhs
downstream
Up-R
Full-R
Down-R
HPH-F
HPH
HPH-R
nest-F
upstream
HPH-P-F
downstream
HPH
nest-R
HPH-P-R
Modified genome
I-F
upstream
HPH
downstream
I-R
