# Supplementary material for: Hydroxybenzoate hydroxylase genes underlying protocatechuic acid production in Valsa mali are required for full pathogenicity in apple trees
Source: Mol Plant Pathol. 2021 Aug 13;22(11):1370–82. doi: 10.1111/mpp.13119 (PMC8518569; doi:10.1111/mpp.13119)
Supplement: Supplementary file 6 [file MPP-22-1370-s006.docx]

M WT *ΔVmHbh1* *ΔVmHbh1-C*

2000 bp

1000 bp

EF1-a

M WT *ΔVmHbh2* *ΔVmHbh2-C*

2000 bp

1000 bp

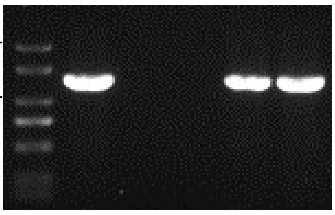

EF1-a

M WT *ΔVmHbh3* *ΔVmHbh3-C*

2000 bp

1000 bp

EF1-a

M WT *ΔVmHbh4* *ΔVmHbh4-C*

3000 bp

2250 bp

EF1-a
